# Analysis of Human Uniparental Embryonic Stem Cells Reveals New Putative Imprinted Loci

**DOI:** 10.1111/cpr.70150

**Published:** 2025-12-02

**Authors:** Shay Kinreich, Nissim Benvenisty

**Affiliations:** ^1^ The Azrieli Center for Stem Cells and Genetic Research, Department of Genetics The Alexander Silberman Institute of Life Sciences, the Hebrew University of Jerusalem Jerusalem Israel

**Keywords:** DNA methylation, epigenetics, genomic imprinting, human embryonic stem cells

## Abstract

Genomic imprinting, an epigenetic process resulting in parent‐specific gene expression, is essential for normal development and growth. Disruption of imprinting leads to various developmental disorders and cancers, yet our understanding of the full repertoire of imprinted genes in humans remains incomplete. Here, we utilised androgenetic, parthenogenetic and biparental human embryonic stem cells and their neural derivatives to identify novel imprinted genes by analysing their methylome and transcriptome profiles. Our analysis revealed 12 novel putative imprinted genes distributed across four distinct loci, with six of them clustered in an uncharacterised imprinted region on chromosome 19. We identified potential imprinting control regions regulating this novel cluster, suggesting a coordinated regulatory mechanism. Notably, these imprinted genes are enriched in cancer‐related pathways, with several showing isoform‐specific imprinting patterns. Our analysis also revealed consistent DNA methylation aberrations in pluripotent stem cells at specific imprinted loci, highlighting potential epigenetic instability during culturing. These findings contribute to our understanding of genomic imprinting regulation in human development and highlight potential genomic regions for further investigation of imprinting‐related disorders.

## Graphical abstract

To identify novel imprinted genes, parthenogenetic, androgenetic and biparental human embryonic stem cells and their differentiated neural progenitors were analysed by methylome and transcriptome profiling. This approach uncovered 12 putative novel imprinted genes, including a clustered region on chromosome 19, expanding the current catalogue of genomic imprinting in humans.

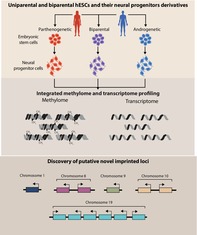

## 1 Introduction

Genomic imprinting is an epigenetic process causing parent‐specific gene expression crucial for growth and development [1, 2]. Imprinted genes regulate fetal development, with errors in imprinting in humans linked to developmental disorders such as Prader‐Willi and Angelman syndromes [3, 4]. Parental imprinting is also related to cancer [5], where loss of imprinting (LOI) in tumour‐suppressor or growth‐regulatory functions may lead to abnormal cell proliferation.

Imprinting is established through parent‐specific DNA methylation ending up in differential methylated regions (DMRs), originating in oocytes and sperm and maintained through fertilisation and early development [1]. These germline DMRs (gDMRs) guide mono‐allelic expression patterns, while secondary DMRs are established after implantation to reinforce imprinting in certain tissues [1]. They typically form as a consequence of mono‐allelic expression that was initially established by gDMRs or other regulatory factors. Imprinted expression can also vary by tissue or be specific to certain gene isoforms, adding complexity to the regulatory landscape [6]. There are over 70 known imprinted genes in human [7].

Discovering new imprinted genes may potentially provide new insights into developmental biology and reveal key regulatory mechanisms in growth and tissue differentiation. These insights could advance knowledge of genetic diseases linked to imprinting errors and offer novel therapeutic targets, especially in personalised medicine for imprinting‐related disorders and certain cancers where gene regulation is disrupted.

Multiple genome‐wide approaches for identifying imprinted genes in humans were conducted, including allele‐specific expression (ASE) and DNA methylation analysis, which detect differences in gene expression and methylation between maternal and paternal alleles [8, 9, 10, 11]. While these approaches are valuable, they rely on one method—either expression or methylation—to identify imprinted genes, which can lead to incomplete or biased detection of the full repertoire of imprinted genes in the human genome.

Imprinting analysis in human pluripotent stem cells (PSCs) has emerged as a powerful approach to identify imprinted genes. Human PSCs, including human embryonic stem cells (hESCs) and induced pluripotent stem cells (iPSCs), maintain their genomic imprints during self‐renewal and can recapitulate tissue‐specific imprinting patterns upon differentiation, making them valuable models for studying imprinting regulation during early embryonic development [12]. In PSCs, imprinting marks are generally stable, though some studies have reported sporadic LOI during prolonged culture, particularly at specific loci such as *H19*/*IGF2* and *DLK1*‐*DIO3* [13], highlighting the importance of regular monitoring of imprinting status in these cells.

Uniparental hESCs have provided unique insights into parental‐specific gene regulation and imprinting mechanisms by allowing the study of exclusively maternal or paternal genomes. Parthenogenetic hESCs (pESCs) can be generated from an oocyte that develops into a blastocyst without fertilisation, leading to the expression of only maternal genes [14, 15], alternatively, parthenogenetic iPSCs can be created by reprogramming of parthenogenetic teratoma [16]. Androgenetic hESCs (aESCs) are derived from the introduction of a sperm into an enucleated oocyte, resulting in nuclear genetic material only from the paternal origin [17, 18]. Both parthenogenetic and androgenetic PSCs can differentiate into all three germ layers [18].

By isolating DNA methylation and gene expression from paternal or maternal alleles in androgenetic and pESCs, it becomes possible to differentiate the unique contributions of each allele to the overall gene expression landscape. This approach enables the identification of imprinted genes with greater specificity, as it highlights allele‐specific regulatory mechanisms that may otherwise remain obscured in biparental cells [18]. Furthermore, studying hESCs enhances the relevance of the findings across differentiated tissues, as these cells can give rise to multiple lineages.

Here, we utilised parthenogenetic, androgenetic and biparental hESCs to identify novel imprinted genes, by comparing comprehensive methylome and transcriptome data in undifferentiated PSCs and their differentiated neural cells. Our analysis revealed 12 novel putative imprinted genes distributed across four distinct loci. By identifying novel putative imprinted genes and characterising their regulatory mechanisms, this work adds to the growing body of knowledge on genomic imprinting in humans and may provide insights into developmental processes influenced by parent‐of‐origin effects.

## 2 Results

### 2.1 Analysis of Uniparental and Biparental Human Embryonic Stem Cells and Their Neural Derivatives

**FIGURE 1:**
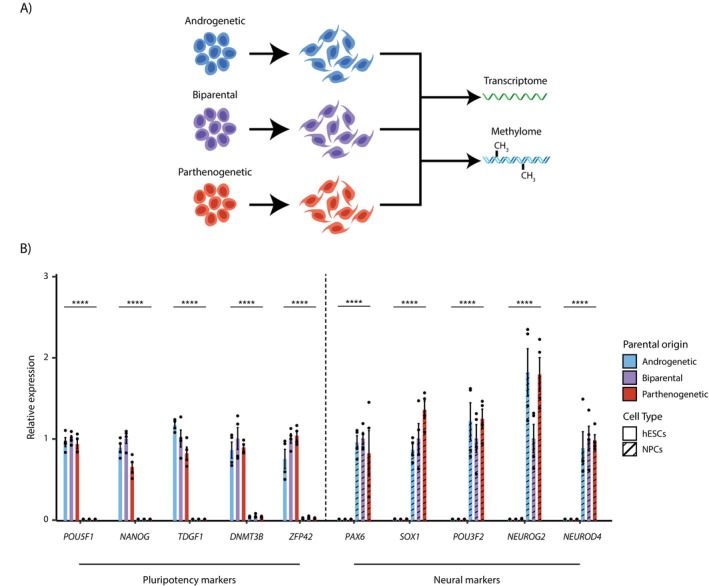
Differentiating uniparental and biparental human embryonic stem cells into neural progenitor. (A) Schematic illustration depicting in vitro differentiation of androgenetic, biparental and parthenogenetic human embryonic stem cells (hESCs) into neural progenitor cells (NPCs) followed by the collection of transcriptome and methylome data. (B) Barplot representing relative transcript levels of pluripotency and neural markers normalised to expression of the relevant biparental cell type of each marker (mean ± SEM values, *n* = 4 [two biological replicates of two cell lines], ****FDR < 0.0001, significance shown between cell types of the same parental origin). See also Figure S1.

To identify novel imprinted genes using uniparental hESCs, we generated methylome data from androgenetic, biparental and parthenogenetic hESCs (Figure 1A). For this, two lines of each parental origin were used: aES3 and aES5 for aESCs, CSES2 and CSES7 for biparental hESCs (ESCs) and pES10 and pES12 for pESCs. To expand the analysis, we differentiated each cell line into neural progenitor cells (NPCs) using dual SMAD‐inhibitor protocol [19] and generated methylome data for the NPCs as well. Including analysis of methylome data of NPCs provided an important validation step to ensure that the identified imprinted DMRs are not limited to a single cell type. Because imprinted DMRs are expected to retain differential methylation across multiple developmental contexts, consistent patterns observed in both ESCs and NPCs strengthen the evidence that these regions represent genuine imprinted loci rather than cell type‐specific epigenetic features. As imprinted DMRs contain multiple CpGs, we employed reduced representation bisulfite sequencing (RRBS) method, which enriches for CpG islands [20]. In order to compare the methylation status of the genes with their expression pattern, we extracted RNA from each of the uniparental and biparental hESCs and their differentiated NPCs. Next generation RNA sequencing (RNA‐seq) was performed on all samples (Figure 1A).

We first assessed NPCs differentiation by analysing the RNA‐seq, both at the global transcriptome levels and through the expression of specific gene markers. The global transcriptome levels revealed significant divergence between hESCs and their differentiated NPCs, while clustering tightly between cells of different parental origin (Figure S1A). We also performed separate PCA plots for each differentiation stage. The analysis showed no apparent clustering between uniparental and biparental hESCs or NPCs, indicating that the global transcriptome differences are predominantly driven by the differentiation state rather than parental origin (Figure S1B,C). Expression of specific gene markers showed significant downregulation of pluripotency markers after differentiation, while neural progenitor markers, such as *PAX6* and *SOX1*, were significantly upregulated (Figure 1B). Gene set enrichment analysis demonstrated that hESCs from different parental origins exhibited concordant enrichment patterns, with the same gene sets being upregulated and downregulated (Figure S1D). In addition, FACS analysis using an antibody against NCAM1 confirmed nearly complete differentiation, with almost 100% of cells expressing NCAM1 (Figure S1E), adding further support to the notion that the cells differentiated toward a similar cellular identity.

**FIGURE 2:**
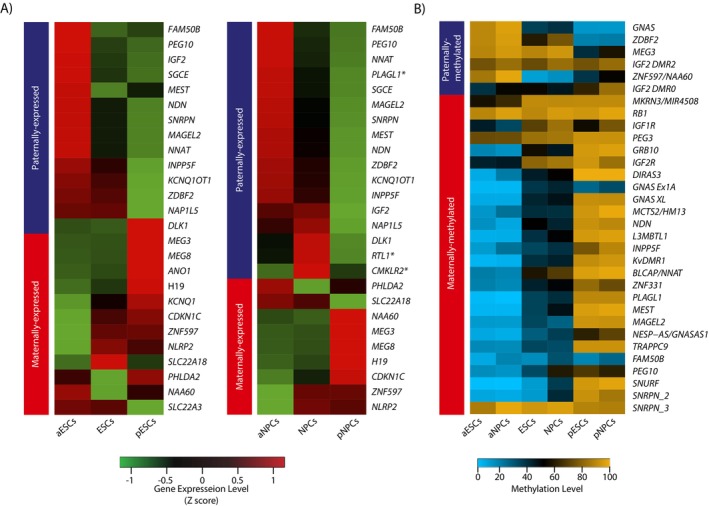
Imprinting status of known imprinted loci in uniparental and biparental hESCs. (A) Heatmaps representing expression in uniparental and biparental hESCs of known imprinted genes which exceed TPM of 1 in either hESCs (left) or in NPCs (right. Asterisk marks genes that are expressed only in NPCs). (B) Heatmap representing mean DNA methylation in known imprinted loci in uniparental and biparental hESCs and NPCs.

Next, we assessed the validity of the different paternal origin hESCs and validated their imprinted behaviour. Known paternally expressed genes (PEGs) were generally upregulated in aESCs and downregulated in pESCs, while maternally expressed genes (MEGs) showed the opposite trend (Figures 2A and S1D). This trend was maintained in NPCs and extended to genes that were not expressed in hESCs, such as *PLAGL1* and *RTL1* (Figures 2A and S1F), suggesting that when looking for novel imprinted genes by expression, the specific tissue being explored is critical, and that the genes in question must be expressed in that tissue. Notably, we observed that *DLK1*, which typically exhibits paternal expression, showed abnormal maternal expression in hESCs, consistent with previous findings [18], while recovering its expected paternal expression pattern in neuronal cells.

Methylation levels of the known imprinted DMRs were generally consistent with the parental origin, with paternally methylated DMRs hypermethylated in androgenetic and hypomethylated in parthenogenetic (Figure 2B). Interestingly, several DMRs showed hypermethylation in all cell types, namely *MEG3*, *IGF2*‐*DMR2*, *IGF2*‐*DMR0*, *MKRN3*/*MIR4508*, *RB1*, *PEG3* and *SNRPN_3*. Notably, *IGF2*‐*DMR0* was less hypermethylated compared to the other DMRs, although it was still abnormally hypermethylated. These DMRs formed two groups: one where a significant difference between androgenetic and parthenogenetic cells persisted, specifically *MEG3*, *IGF2*‐*DMR0* and *MKRN3*/*MIR4508*, and another where the DMRs were uniformly hypermethylated across all samples, including *IGF2‐DMR2*, *RB1*, *PEG3* and *SNRPN_3*.

### 2.2 Consistent DNA Methylation Aberrations in Known Imprinted Loci in Biparental Cells

To further investigate this phenomenon, we downloaded more than 140 methylome samples from the Sequence Read Archive (SRA) database and categorised them into three groups: pluripotent cells and their differentiated derivatives, fetal samples and adult samples. Each sample was included in the analysis only if at least half of the known DMRs had normal methylation levels (ranging from 30 to 70), resulting in a final dataset of 75 samples (Table S1).

Our analysis revealed that the DMRs hypermethylated in our samples are also hypermethylated in PSCs from different origins, including iPSCs and hESCs lines such as WA01 and WA09, and their differentiated derivatives. In contrast, fetal and adult samples generally exhibit around 50% methylation levels (Figure S2A). Further analysis of the CpGs in each sample indicated that in hypermethylated samples almost all of the CpGs within the DMRs are hypermethylated, both for DMRs without differences between uniparental hESCs samples, such as *RB1*, and for those demonstrating differences, such as *MEG3* (Figure S2B).

The phenomenon of LOI in iPSCs is well documented [13] and is generally attributed to their somatic origin, often assessed through allelic expression analysis [21, 22]. Our findings highlight specific aberrations in DMRs across multiple PSCs from diverse origins, suggesting an underlying aberration mechanism in these regions. Given these observations, we chose to focus primarily on the uniparental cells in our analysis, while still including the biparental cells for reference.

### 2.3 Identification of Novel Putative Imprinted Genes Using Uniparental and Biparental hESCs and NPCs

**FIGURE 3:**
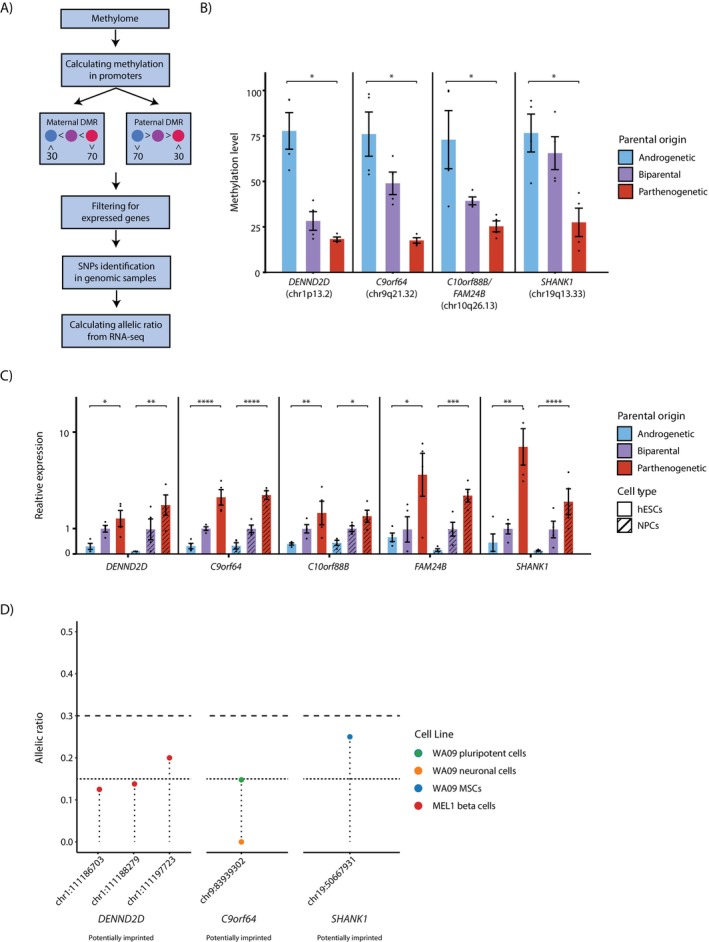
Identification of novel putative imprinted genes using uniparental and biparental hESCs and NPCs. (A) Schematic overview of the bioinformatic analysis used to identify new putative imprinted genes. (B) Barplot showing the average DNA methylation levels of five promoters (spanning −500 to +500 base pairs from the transcription start site) which exhibit imprinted patterns (mean ± SEM values, *n* = 4 (two hESC samples and two NPC samples), *FDR < 0.05, Wilcoxon test). (C) Barplot displaying relative expression levels of the potentially imprinted genes, normalised to expression in their corresponding biparental cell types, in hESCs or NPCs (mean ± SEM values, *n* = 4 [two biological replicates of two cell lines], *FDR < 0.05, **FDR < 0.01, ***FDR < 0.001, ****FDR < 0.0001). (D) Allelic ratio of SNP expression of the potentially imprinted genes. Points below the dotted line represent monoallelic expression. Points between the dotted and the dashed lines represent partially monoallelic expression (*n* = 3–63, > 5 reads for each gene). See also Figure S3.

Using our unique system of uniparental hESCs, we sought to identify previously uncharacterised imprinted genes. We began by analysing the methylome, focusing on genes with DMRs in their promoters, defined as the region spanning −500 to +500 base pairs from the transcription start site (TSS). Specifically, we compared the promoter DNA methylation profiles between androgenetic, biparental and parthenogenetic samples to identify genes exhibiting differential methylation, potentially indicating novel imprinted loci. Even though some known DMRs show consistent DNA methylation aberrations in biparental cells, most of the known DMRs exhibit normal behaviour. We included the biparental cells to highlight their relationship with the uniparental hESCs and provide a reference for future studies of these loci. However, they were not included in the bioinformatic analysis for detection of novel DMRs. We focused on genes that are expressed in hESCs or NPCs (Figure 3A).

Unbiased promoter analysis revealed six expressed genes with significant paternal DMRs in their promoters (Figures 3B and S3A–C). Of these, five genes, namely *DENND2D*, *C9orf64*, *C10orf88B*, *FAM24B* and *SHANK1*, showed expression patterns consistent with their promoter DMRs, displaying significantly higher expression in the parthenogenetic samples compared to the androgenetic samples (Figure 3C). In agreement with these results, the expression of these genes in biparental cells was lower than that in parthenogenetic cells and higher than in androgenetic cells (Figures 3C and S3D–E). However, one gene, *SHARPIN*, that showed differential methylation between the parthenogenetic and the androgenetic cells, exhibited similar expression levels across the uniparental and biparental samples (Figure S3F), suggesting that its regulation may be isoform‐specific, with particular isoforms being differentially regulated depending on the parental origin, while others remain unaffected by the methylation changes observed [6].

Identifying monoallelic expression provides an additional layer of support for confirming a gene as imprinted. In our biparental hESCs, the RRBS revealed no informative SNPs in the putative imprinted genes, necessitating the use of publicly available datasets with heterozygous SNPs to confirm ASE. To do this, we downloaded from the SRA database genomic samples of various hESCs, identifying all samples with informative single nucleotide polymorphism (SNPs) in the candidate imprinted genes. We then examined all corresponding RNA samples to analyse the allelic ratio at the SNP site, searching for monoallelic expression, including all datasets with informative SNPs, regardless of their allelic expression (Figure 3A). We conducted SNP analysis and identified informative SNPs in the *DENND2D* gene in the MEL1 cell line, in *C9orf64* and *SHANK1* genes in the WA09 cell line and in *SHARPIN* in RUES2 and WA01 cell lines. For every gene we calculated the allelic ratio in different samples, as a proportion of RNA reads corresponding to the allele with the least expression at a specific SNP site relative to the total number of reads at that site. Since imprinted genes can exhibit isoform‐specific imprinting, it is possible for some tissues to express both alleles (biallelic expression) for certain isoforms while maintaining monoallelic expression for others. This variation in isoform expression can result in SNPs showing biallelic expression, even in imprinted regions. Therefore, we focused on cell types that express these genes, displaying monoallelic SNP expression, where, according to ‘The International Stem Cell Initiative’, an allelic ratio below 0.15 is considered monoallelic expression, and an allelic ratio between 0.15 and 0.3 is considered partially monoallelic expression, a characteristic pattern that imprinted genes often display as well [12]. Our analysis revealed that SNPs in *DENND2D* exhibit monoallelic and partially monoallelic expression in cells differentiated into beta cells. SNPs in *C9orf64* show monoallelic expression in both pluripotent and neuronal cells, while SNPs in *SHANK1* exhibit partially monoallelic expression in mesenchymal stem cells (MSCs) (Figures 3D and S3G). SNPs in *SHARPIN* show monoallelic expression in osteoblasts differentiation and partially monoallelic expression in pluripotent cells (Figure S3H).

It is important to notice that allelic bias in gene expression is a widespread phenomenon in mammalian cells, with studies demonstrating that a substantial fraction of autosomal genes (~5%–10%) exhibit random monoallelic expression [23]. To distinguish genuine genomic imprinting from this background of random allelic bias, our study employed a dual‐evidence approach that goes beyond allelic analysis alone. We required convergent evidence through coordinated uniparental‐specific expression and DNA methylation patterns at putative DMRs, which provide a critical epigenetic signature that distinguishes imprinted genes from those showing random allelic bias. The combination of parent‐of‐origin‐specific expression and methylation differences and coordinated allelic expression provides substantially stronger evidence for genuine genomic imprinting than expression analysis alone, ensuring that our identified candidates represent authentic putative imprinted loci rather than genes with stochastic expression patterns.

### 2.4 Identification of New Putative Imprinted Loci at Chromosomes 8 and 19

Imprinted genes are often organised in clusters, as seen in well‐known imprinted loci such as the *H19*‐*IGF2* and Beckwith‐Wiedemann locus on chromosome 11, and the Prader‐Willi locus on chromosome 15 [24, 25]. To identify potential imprinted genes located near the novel imprinted genes we discovered, we conducted a search within a 1 Mb window around the TSSs of these genes. This search was based on more relaxed criteria than the initial analysis, requiring either imprinted‐like expression patterns or the presence of promoter DMRs. While the first analysis focused on genes with very stringent criteria, this approach allowed us to capture additional candidates where a DMR in one imprinted gene may influence the expression of nearby genes, or where genes with nonimprinted expression may still be regulated by imprinting mechanisms, potentially exhibiting tissue‐ or isoform‐specific expression.

**FIGURE 4:**
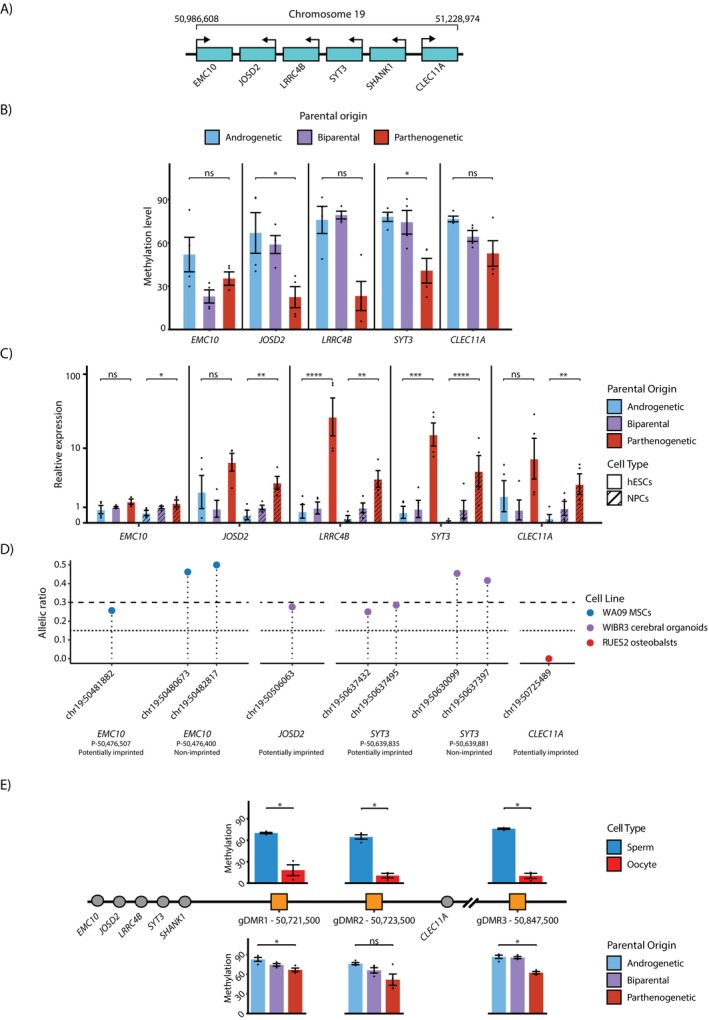
Identification of new putative imprinted locus at chromosome 19. (A) Schematic illustration of the new putative imprinted locus at chromosome 19. (B) Barplot showing the average DNA methylation levels of five promoters of potentially imprinted genes on chromosome 19 (mean ± SEM values, *n* = 4 [two hESC samples and two NPC samples], ns = not significant, *FDR < 0.05, Wilcoxon test). (C) Barplot displaying relative expression levels of potentially imprinted genes on chromosome 19, normalised to expression in their corresponding biparental cell types, in hESCs and NPCs (mean ± SEM values, *n* = 4 [two biological replicates of two cell lines], ns = not significant, *FDR < 0.05, **FDR < 0.01, ***FDR < 0.001, ****FDR < 0.0001). (D) Allelic ratio of SNP expression of the additional potentially imprinted genes on chromosome 19. Points below the dotted line represent monoallelic expression. Points between the dotted and the dashed lines represent partially monoallelic expression. Note that for both *EMC10* and *SYT3* genes there is an isoform that is potentially imprinted and an isoform that is not imprinted (*n* = 3–63, > 5 reads for each gene). (E) Schematic illustration of the suggested germline DMRs (gDMRs) at chromosome 19. Barplots above the illustration show average DNA methylation of the gDMRs in germ cells (*n* = 4 for sperm, *n* = 3 for oocyte, *FDR < 0.05, obtained by permutation test). Barplots below the illustration show average DNA methylation of the gDMRs in uniparental and biparental hESCs (mean ± SEM values, *n* = 4 [two hESC samples and two NPC samples], ns = not significant, *FDR < 0.05). See also Figure S4.

On chromosome 19 we identified five genes with promoter DMR and/or imprinted‐like expression in proximity to *SHANK1*: *EMC10*, *JOSD2*, *LRRC4B*, *SYT3* and *CLEC11A* (Figure 4A). Among them, two genes (*JOSD2* and *SYT3*) have clear DMR, with significant differential methylation levels between the uniparental cells (Figures 4B and S4A), whereas all five genes exhibit imprinted‐like expression, whether in NPCs only or in both hESCs and NPCs (Figures 4C and S4B). While most identified genes exhibit pronounced differences in expression and methylation between androgenetic and biparental cells, some loci show more variable patterns. This variability is consistent with our observations that genomic imprinting can be incompletely maintained in cultured biparental hESCs, like the behaviour of the well‐characterised *MEG3* DMR (Figure 2B), leading to partial LOI at specific loci. SNP analysis revealed partially monoallelic expression of *EMC10* in MSCs, and of *JOSD2* and *SYT3* in cerebral organoids, with *CLEC11A* displaying monoallelic expression in osteoblasts (Figure 4D). Notably, our analysis identified at least two isoforms for *EMC10* and *SYT3*, with only one of them being imprinted, suggesting that these two genes are isoform‐specific imprinted genes (Table S2).

Imprinting is established during germ cells formation through the creation of DMRs between sperm and oocyte, which persist through DNA methylation reprogramming process after fertilisation, known as gDMRs. Some gDMRs function also as imprinting control regions (ICRs) which can establish secondary DMRs after implantation [26], as secondary DMRs typically form as a consequence of mono‐allelic expression that was initially established by gDMRs. To identify potential gDMRs on chromosome 19, that persist after fertilisation and thus could potentially be an authentic gDMRs, we analysed DNA methylation data of sperm and oocyte. All novel DMRs on chromosome 19 were found to be secondary DMRs. To locate possible gDMRs in this locus we divided the genomic region 200 Kb centromeric to *EMC10* and 200 Kb telomeric to *CLEC11A* into windows of 1 Kb and analysed their DNA methylation levels in sperm and oocyte, without being limited to annotated promoters, enabling the discovery of previously undetected DMRs that may have been missed in our initial analysis. We identified three putative gDMRs, two of them are located between *SHANK1* and *CLEC11A* separated by a 1 Kb gap, which was not covered by the RRBS, and a third lies ~120 Kb bases telomeric to *CLEC11A* (Figure 4E and Table S3). These DMRs in germ cells were partially persisted in uniparental hESCs, showing significant differences between parthenogenic and androgenetic cells in two of the three gDMRs.

On chromosome 8, adjacent to *SHARPIN*, we found the gene *WRD97* as having a promoter DMR (Figure S4C). However, even though a slight increase in expression was noted in the androgenetic cells, the gene did not display significant imprinted‐like expression, suggesting it may be a tissue‐ or isoform‐specific imprinted gene (Figure S4D). SNP analysis found that the WA01 cell line contains four SNPs for this gene, and in neuronal samples sufficient coverage showed the expression of at least two isoforms: one with complete or partial monoallelic expression and the other isoform with biallelic expression. This dual isoform expression likely suggests an isoform‐specific imprinted gene (Figure S4E).

### 2.5 Synteny of the Novel Putative Imprinted Genes in Mouse

Even though some imprinting is conserved between human and mouse, particularly among the first imprinted genes discovered, only 20%–35% of imprinted genes in humans are also present and imprinted in mice [27, 28]. Notably, these conserved imprinted genes typically retain their syntenic clustering across species, maintaining their genomic organisation and relative positioning.

We focused our investigation on the putative imprinted genes located on human chromosome 19. Through detailed synteny mapping, we identified that the genomic region chr7: 43,179,022–43,458,159 in mouse, exhibits 80% synteny to the genomic site of the putative imprinted cluster on chromosome 19 in humans. To search for imprinting in the mouse orthologous region, we conducted methylation analysis to examine the methylation status of the syntenic site of the gDMRs on chromosome 19 and detected corresponding gDMRs in the mouse orthologous region for two of the gDMRs in humans (Figure S4F).

Additionally, we employed strain‐specific SNP analysis using crosses between genetically distinct mouse strains, Cast/EiJ (paternal) × C57BL/6J (maternal). This approach allowed us to track the parental origin of gene expression by identifying which parental allele was being expressed in the offspring. RNA‐seq reads were mapped to the C57BL/6J reference genome, and ASE was quantified by determining the ratio of reads mapping to strain‐specific SNPs. This analysis provided direct evidence of maternal expression for two of these putative imprinted genes on chromosome 19. Both genes exhibited alternative allele ratios below 0.3, indicating predominant expression from the maternal allele and confirming parent‐of‐origin‐dependent gene expression (Figure S4G).

## 3 Discussion

In this study, we utilised the transcriptome and methylome of uniparental and biparental hESCs to identify 12 novel putative imprinted genes distributed across four distinct loci, providing valuable insights into the regulation of genomic imprinting. Notably, one major locus on chromosome 19 contained six of these newly identified putative imprinted genes, suggesting that this region may play a central role in imprinting regulation. Moreover, our analysis highlighted the potential presence of gDMR in this region, which might contribute to the regulation of the parent‐specific expression of these genes. Interestingly, our analysis identified both MEGs and PEGs. However, all the PEGs identified were already well‐characterised in previous studies, leaving the MEGs as the novel imprinted genes. This suggests that earlier approaches for identifying imprinted genes in humans may have been limited in their ability to detect MEGs, potentially due to limitations in genome‐wide methylation and expression analyses.

In our previous work [18], we analysed DNA methylation in hESCs, aESCs and pESCs using the Illumina 450 K methylation array, which covers approximately 450,000 CpG sites throughout the genome. In the current study, we employed RRBS, which provides coverage of roughly 80% of CpG islands and allowed us to analyse more than 4.5 million CpG sites. This 10‐fold increase in coverage, combined with the enhanced resolution and sensitivity of sequencing‐based methods compared to array technology, enabled the detection of novel putative imprinted regions that were not captured in our previous work. The improved dynamic range of RRBS also facilitated the identification of regions with more subtle parent‐of‐origin methylation differences, particularly at loci exhibiting isoform‐specific patterns.

Our analysis of DNA methylation patterns in PSCs revealed distinct hypermethylation at several DMRs, suggesting potential disruption of normal imprinting regulation during culturing and cellular reprogramming. This observation aligns with previously reported LOI of *IGF2* and *MEG3* in PSCs [21]. The hypermethylation patterns we observed were notably consistent across multiple DMRs, indicating a systematic rather than random alteration of methylation marks during the establishment and maintenance of pluripotency. This phenomenon may arise from both biological and culture‐related effects. These regions might contain sequence elements that strongly attract DNA methyltransferases regardless of parental origin; they could be associated with specific chromatin modifiers that maintain their methylated state during cell divisions; or they might serve as binding sites for pluripotency factors that influence local epigenetic states in stem cells, as was shown for the *IGF2/H19* locus [29]. Culture‐related factors, such as cellular stress, media composition or oxygen tension, may also explain these aberrant methylation patterns. A comprehensive way to determine whether DMR hypermethylation arises during line establishment or accumulates in culture would be to perform longitudinal methylome profiling across cell passages starting from cell line establishment, combined with analyses of ASE and controlled variation of culture conditions. This finding has crucial implications for the use of PSCs in both research and therapeutic applications, as it suggests that current methodologies of PSCs' derivation do not fully recapitulate the complex epigenetic landscape of normal development. Furthermore, the correlation between these hypermethylated regions and known LOI genes underscores the importance of maintaining proper methylation patterns for accurate imprinting control. Our results emphasise the need for improved methods to maintain or restore proper imprinting marks in PSCs, particularly when studying parent‐of‐origin effects or developing stem cell‐based therapies where precise regulation of imprinted genes is crucial.

The use of uniparental hESCs as a model system offers significant advantages over previous methods, particularly in detecting subtle imprinting effects that may have been overlooked in biparental systems. In addition, the use of RRBS was key in accurately identifying methylation patterns at imprinted loci, allowing for high‐resolution analysis of DMRs. By analysing both methylation and expression patterns simultaneously, this approach provides a more comprehensive understanding of imprinting dynamics. Furthermore, studying both undifferentiated and differentiated states is crucial because certain genes may only be expressed in one of these stages, and combining methylation and expression data allows for a more accurate identification of imprinted genes. This dual‐stage analysis helps to capture a spectrum of imprinting regulation, revealing genes that might otherwise be overlooked if only one cell state was analysed. In addition, the genes identified as imprinted in our analysis harbour secondary DMRs, which are established after implantation [1]. The identification of putative imprinted genes within these regions suggests that secondary DMRs may be underappreciated in current imprinting studies, and this emphasises the importance of expanding our focus to include these regions when studying imprinting and its potential impact on development and disease. In our initial RNA‐seq and RRBS analyses, we identified 12 novel putative imprinted genes. For three genes no informative SNPs were identified in various hESC samples. Informative SNPs were identified in the remaining nine genes: four had an allelic ratio between 0.15 and 0.3, while five had an allelic ratio below 0.15. To assess the robustness of our ASE analysis, we repeated the analysis using a more stringent read‐depth threshold (≥ 20 reads per SNP site). While our original threshold identified seven putative imprinted genes and two isoform‐specific putative imprinted genes, the more strict cutoff still yielded five putative imprinted genes and one isoform‐specific putative imprinted gene, confirming that our conclusions are not driven by low‐coverage sites. We note, however, that analyses based on public RNA‐seq data may be influenced by dataset‐specific variability, and results should therefore be interpreted as supportive rather than definitive evidence.

Our analysis revealed several genes exhibiting isoform‐specific imprinting patterns, including *EMC10*, *SYT3* and *WRD97*, where certain isoforms show monoallelic expression while others are biallelically expressed. This phenomenon of isoform‐specific imprinting has been previously documented in other genes, such as *GNAS*, where specific transcriptional variants show parent‐of‐origin expression patterns while others escape imprinting control [30]. The identification of isoform‐specific imprinting in our newly discovered genes adds another layer of complexity to genomic imprinting regulation and suggests that this phenomenon might be more widespread than previously recognised. It is possible that other genes identified in our study might also harbour undiscovered isoform‐specific imprinting patterns, particularly given that such patterns can be tissue‐specific or developmentally regulated. This finding highlights the importance of considering transcript‐level analysis in imprinting studies, as gene‐level assessments might mask the intricate regulation of specific isoforms.

Our analysis employed stringent criteria for identifying novel imprinted genes, which successfully led to the discovery of five previously uncharacterised putative imprinted genes. While these strict parameters ensured high confidence in our findings, we acknowledge they may have limited our ability to detect the full spectrum of imprinted genes in the genome. This possibility is illustrated by our subsequent identification of the chromosome 19 putative imprinted cluster, which emerged when we applied less stringent criteria to regions adjacent to all of our confirmed imprinted loci. This approach proved valuable, as proximity to known imprinted genes served as an additional selection criterion that compensated for the relaxed statistical thresholds. The newly identified putative imprinted cluster on chromosome 19 reveals a highly organised structure, with multiple novel putative imprinted genes grouped together in a coordinated regulatory region, in both divergent (head‐to‐head) and convergent (tail‐to‐tail) orientations. This cluster is organised around several putative gDMRs that might play a central role in controlling the unique expression patterns associated with imprinting. The gDMRs within this cluster could function as epigenetic ‘switches’, maintaining the silencing of one parental allele through DNA methylation and histone modifications, which ensures the proper dosage of gene products critical for development. Comparatively, this chromosome 19 cluster exhibits epigenetic similarities to other well‐characterised imprinted clusters, such as those on chromosomes 11 (containing *IGF2* and *H19*) [24] and 15 (containing *SNRPN* and *UBE3A*) [25], both of which are controlled by prominent ICRs that coordinate the imprinting of multiple functionally related genes. Future studies incorporating chromatin conformation data will be important to elucidate how spatial genome architecture contributes to imprinting control in this region.

The clinical significance of our findings is further underscored by studies showing that loss of heterozygosity in the newly identified putative imprinted locus on chromosome 19 is associated with multiple types of human cancer [31, 32, 33]. This finding suggests that proper maintenance of imprinting in this region may play a crucial role in preventing oncogenic transformation across various tissue types. This suggests that disruption of imprinting at this locus might serve as both a diagnostic marker and a potential therapeutic target in cancer treatment, particularly in cases where LOI contributes to disease progression.

The novel putative imprinted cluster on chromosome 19 offers an opportunity to study the interactions between DMRs and nearby genes. Additional experiments, such as chromatin conformation capture or CRISPR‐based epigenetic editing, could elucidate how these regions spatially organise within the nucleus to influence gene expression. Moreover, identifying the transcription factors and noncoding RNAs that interact with the gDMRs may reveal their broader roles in establishing and maintaining imprinting. These studies could significantly advance our understanding of how clustered imprinted genes operate as coordinated units, shedding light on their importance in neurodevelopment and disease pathways.

## 4 Materials and Methods

### 4.1 Cell Lines and Maintenance

The following cell lines were used in this work: aES3, aES5, CSES2, CSES7, pES10 and pES12. The aESC lines (aES3 and aES5) were derived from two different sperm donors [18], while the pESC lines (pES10 and pES12) were derived from oocytes obtained from the same donor [14]. Oocytes from the same donor only share approximately 50% of their genome due to independent meiotic events, and their artificial activation and subsequent maturation occur separately, resulting in independently established methylation landscapes. hESCs were used under the Israeli guidelines concerning hESC research. Cells were thawed and cultured at 5% CO_2_ and 37°C feeder layer mouse embryo fibroblasts (MEFs) in standard hESC growth medium. hESC medium was composed of knockout Dulbecco's modified Eagle's medium (DMEM) supplemented with 15% knockout serum replacement (KSR, Thermo Fisher Scientific), 0.1 mM nonessential amino acids, 2 mM L‐glutamine, 50^−1^ streptomycin, 50^−1^ penicillin, 0.1 mM β mercaptoethanol and 8^−1^ basic fibroblast growth factor (bFGF). MEFs were cultured in DMEM supplemented with 10% fetal bovine serum (FBS, Invitrogen), L glutamine, 50^−1^ streptomycin and 50^−1^ penicillin. All experiments were performed using hESCs at passages 8–15.

### 4.2 Differentiation Into Neural Progenitor Cells

For NPCs differentiation, hESCs were plated on Matrigel‐coated 6‐well plates (Corning) at a density of 2 million cells per well in mTeSR1 (STEMCELL Technologies) supplemented with 10 μM ROCK inhibitor Y‐27632 (Stemgent) for 1 day after splitting. The next day, mTeSR1 medium was replaced by embryoid body (EB) medium (knockout DMEM supplemented with 15% KSR (Thermo Fisher Scientific), 0.1 mM nonessential amino acids, 2 mM L‐glutamine, 50^−1^ streptomycin, 50^−1^ penicillin, 0.1 mM β mercaptoethanol and 8^−1^) supplemented with 10 μM of SB431542 (Biogems) and 2.5 μM of LDN193189 (Biogems). EB medium (3 mL/well) was replaced every 24 h for 4 days. Medium was then changed to a composite medium of EB medium and N‐2 medium (DMEM/Nutrient Mixture F‐12 Ham (Sigma), 1 × N‐2 supplement (Thermo Fisher, cat. no. 17502001), 50^−1^ streptomycin, 50^−1^ penicillin, 2 mM L‐glutamine, 0.1 M ascorbic acid (Sigma) and 0.16% D‐Glucose) at a 3:1 ratio in the presence of 10 μM of SB431542 and 2.5 μM of LDN193189 for 24 h. The next day, medium was changed to a 1:1 ratio mix of EB medium and N‐2 medium in the presence of 10 μM of SB431542 and 2.5 μM of LDN193189 for another 24 h. The following day, the medium was changed to a 1:3 mix of EB medium and N‐2 medium in the presence of 10 μM of SB431542 and 2.5 μM of LDN193189 for another 24 h. Next, the medium was changed to N‐2 medium in the presence of 10 μM of SB431542 and 2.5 μM of LDN193189 for another 24 h and the cells were harvested the following day (9th day) for downstream analyses. Control cells were harvested before the differentiation process and analysed the same way as the differentiated cells. Each cell line was cultured and analysed in duplicate, with each replicate processed independently through culture, differentiation and sample preparation.

### 4.3 DNA Extraction and DNA Methylation Sequencing

Genomic DNA was extracted with Blood & Cell Culture DNA Midi Kit (Qiagen) according to the manufacturer's instructions. Four samples (aES5 and CSES7, both hESCs and NPCs) were sent to CD Genomics for RRBS. The rest of the samples were processed for RRBS using Zymo‐Seq RRBS Library Kit (cat. no. D5461) according to the manufacturer's protocol and sequenced using Illumina NextSeq 500. Results were analysed by performing adapters' sequences trimming using the TrimGalore tool and the reads were aligned, and per‐base methylation metrics were extracted based on the reference genome (GRCh38) using the Bismark tool. As hESCs samples were originally grown on MEFs, reads originating from mouse cells were filtered out from each sample using the XenofilteR software [34].

### 4.4 RNA Isolation and RNA Sequencing

Total RNA was isolated using the RNeasy Mini Kit (Qiagen) and the mRNA fraction of total RNA was enriched by pulldown of poly(A) RNA. RNA‐seq libraries were generated using the TruSeq RNA Library Prep Kit (Illumina) according to the manufacturer's protocol and sequenced using the Illumina NextSeq 500 with 76 bp single‐end reads. Reads were mapped to the GRCh38 reference genome using STAR [35]. Reads originating from mouse cells were filtered out from each sample using the XenofilteR software [34]. Normalisation of the read counts, differential expression and statistical analysis were performed using the EdgeR package in R [36], generating CPM values. Values of biological repeats were averaged. A gene was considered expressed if it remained in the CPM table after regular EdgeR filtration. Differential expression analysis was done using a generalised linear model approach. GO analysis was performed using the GSEA tool [37, 38]. Enriched GO terms were considered significant if their FDR score was lower than 0.05.

### 4.5 Flow Cytometry

Differentiated cells were stained and analysed for a marker of NPC differentiation. The cells were dissociated using TrypLE Select (Thermo Fisher Scientific) into single cells and centrifuged at 150 g at 4°C. Cells were washed with ice‐cold 10% FBS‐PBS twice, and incubated with goat antihuman/mouse NCAM1 antibody (R&D Systems, cat. no. AF2408) diluted 1:40 in 10% FBS‐PBS for 1 h. Cells were washed with ice‐cold 10% FBS‐PBS twice, incubated with donkey antigoat Cy5‐conjugated secondary antibody (Jackson ImmunoResearch Laboratories) diluted 1:200 in 10% FBS‐PBS for 1 h and then washed again with ice‐cold 10% FBS‐PBS twice. At the end of staining, cells were filtered through a 70 μm cell strainer and analysed by flow cytometry (BD Biosciences FACSAria III) and Flowjo software (FlowJo LLC).

### 4.6 Promoter Methylation Analysis

Promoter DNA methylation was analysed to identify imprinted loci by focusing on the region spanning −500 to +500 base pairs from the TSS of each gene. Methylation levels were quantified using the CpG site‐specific outputs generated by the Bismark tool. Differential methylation between androgenetic, biparental and parthenogenetic samples was assessed. Genes with significant methylation differences in this promoter region, particularly those consistent with parent‐of‐origin methylation patterns, were selected as potential imprinted loci.

### 4.7 Identification of gDMRs on Chromosome 19

To identify potential gDMRs within the imprinted locus on chromosome 19, DNA methylation data sets of oocyte and sperm samples were obtained from GSE49828 and GSE154762 accession numbers and were analysed. The genomic region spanning 200 kb centromeric to EMC10 and 200 kb telomeric to CLEC11A was divided into 1000 bp windows. Average methylation levels within each window were calculated for both oocyte and sperm samples. DMRs were identified as windows exhibiting significant differences in methylation between the two gametes. Candidate gDMRs were defined as DMRs that existed in oocyte vs. sperm samples and persisted in uniparental hESCs, showing differential methylation between androgenetic and parthenogenetic samples.

### 4.8 SNP Analysis

Samples were downloaded from NCBI's SRA and processed using the EpiTyping pipeline [39] to generate ASE files. ASE was assessed at heterozygous SNPs within individual samples, measuring the relative expression of maternal and paternal alleles. Since both alleles are sequenced within the same RNA‐seq library and sequencing run, batch effect correction was not applied, as technical variation affects both alleles equally and does not bias allelic ratios [40]. To minimise technical artefacts, the EpiTyping pipeline incorporates quality control and filtering procedures based on the GATK toolkit, excluding low‐quality and duplicate reads. For several genes consistent monoallelic expression was observed across multiple SNPs and independent datasets, minimising the likelihood of technical artefacts. Sample size for the different cell types was *n* = 63 for WA09 pluripotent cells, *n* = 7 for WA09 neuronal cells, *n* = 9 for WA09 MSCs, *n* = 27 for WA01 pluripotent cells, *n* = 15 for WA01 neuronal cells, *n* = 6 for WIBR3 cells and *n* = 3 for MEL1 and RUES2 samples. Samples accession codes are summarised in Table S4.

### 4.9 Analysis of Mouse Samples

For SNP analysis, mouse hybrid samples were obtained from GSE70484 accession number and were processed using the EpiTyping pipeline, generating ASE files. For methylation analysis, samples were obtained from GSE122829, GSE238227 and GSE240712 accession numbers and were analysed by performing adapters' sequences trimming using the TrimGalore tool and the reads were aligned, and per‐base methylation metrics were extracted based on the reference genome (GRCm39) using the Bismark tool.

## Author Contributions

S.K. and N.B. designed the experiments and interpreted the data. S.K. performed the experiments and analysed the data. S.K. and N.B. wrote the manuscript. N.B. supervised the study.

## Funding

This work was supported by the Azrieli Foundation, the Rosetrees Trust, the Israel Science Foundation (2054/22, 3605/21), the United States‐Israel Binational Science Foundation (2021278) and the HORIZON EUROPE Health (101056712).

## Ethics Statement

All experiments were performed according to the ethical guidelines of the Hebrew University.

## Conflicts of Interest

The authors declare no conflicts of interest.

## Supporting information


**Figure S1:** Additional characterisation of uniparental and biparental hESCs during neural.
**Figure S2:** Consistent DNA methylation aberrations in known imprinted loci in biparental cells.
**Figure S3:** Identification of imprinted genes using uniparental and biparental hESCs and NPCs.
**Figure S4:** Identification of imprinted locus at chromosome 8.
**Table S2:** List of the putative imprinted genes and their gene ID.

**Table S1:** cpr70150‐sup‐0002‐TableS1.csv.

**Table S3:** cpr70150‐sup‐0003‐TableS3.csv.

**Table S4:** cpr70150‐sup‐0004‐TableS4.csv.

## Data Availability

The reduced representation bisulfite sequencing data presented in this article is available in ArrayExpress (accession number: E‐MTAB‐14634). The RNA sequencing (RNA‐seq) data of the library is available in E‐MTAB‐14633. All other data that support the findings of this study are available within the article and its Supporting Information.
