# Supplementary material for: Analysis of Human Uniparental Embryonic Stem Cells Reveals New Putative Imprinted Loci
Source: Cell Prolif. 2025 Dec 2;59(6):e70150. doi: 10.1111/cpr.70150 (PMC13241825; doi:10.1111/cpr.70150)
Supplement: Supplementary file 1 — Figure S1: Additional characterisation of uniparental and biparental hESCs during neural. Figure S2: Consistent DNA methylation aberrations in known imprinted loci in biparental cells. Figure S3: Identification of imprinted genes using uniparental and biparental hESCs and NPCs. Figure S4: Identification of imprinted locus at chromosome 8. Table S2: List of the putative imprinted genes and their gene ID. [file CPR-59-e70150-s001.pdf]

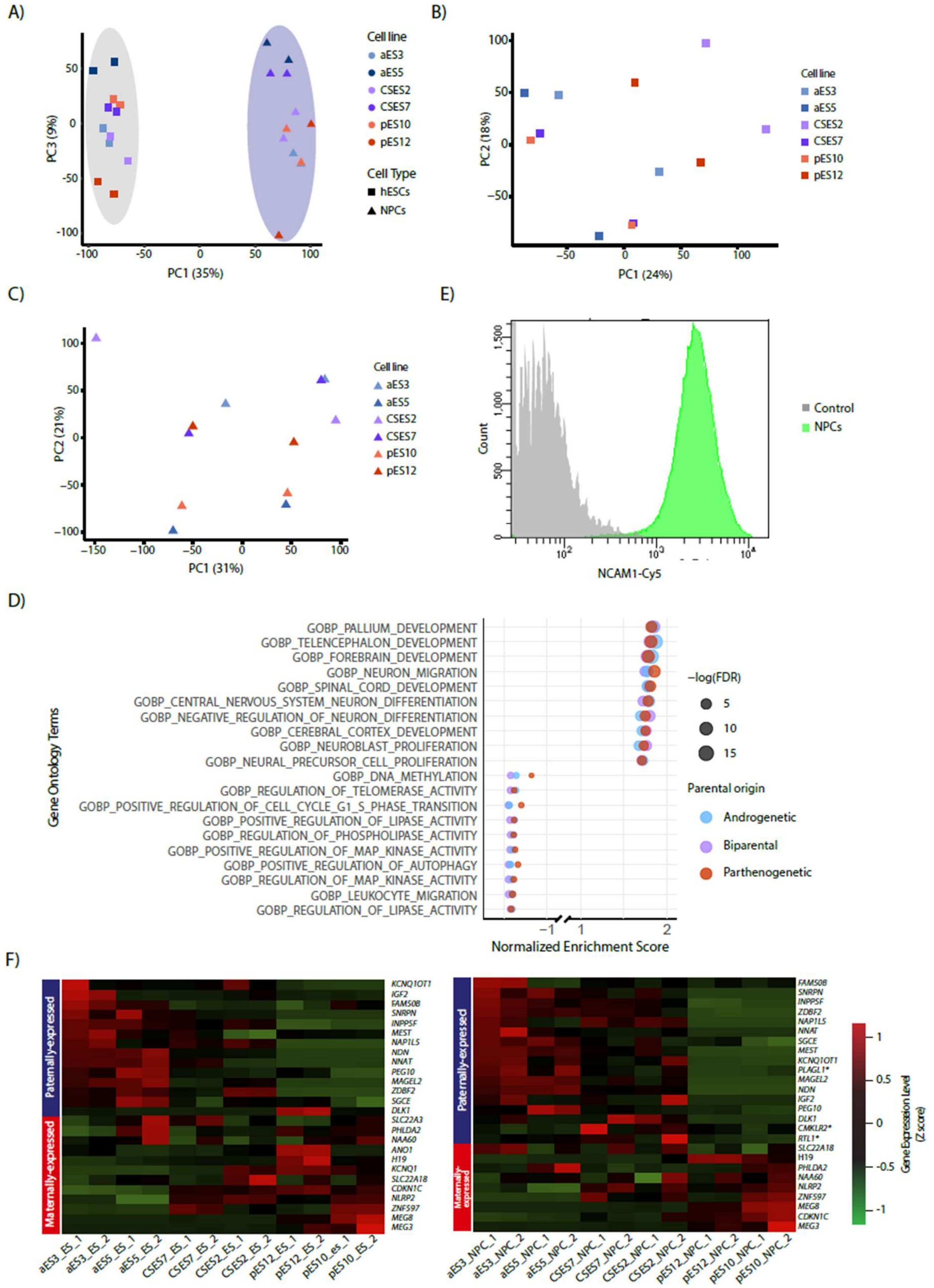

A)
Cell line
aES3
aES5
CSES2
CSES7
pES10
pES12
Cell Type
hESCs
NPCs
PC1 (35%)
PC3 (9%)
B)
Cell line
aES3
aES5
CSES2
CSES7
pES10
pES12
PC1 (24%)
PC2 (18%)
C)
Cell line
aES3
aES5
CSES2
CSES7
pES10
pES12
PC1 (31%)
PC2 (21%)
E)
Control
NPCs
Count
NCAM1-Cy5
D)
GOBP_PALLIUM_DEVELOPMENT
GOBP_TELENCEPHALON_DEVELOPMENT
GOBP_FOREBRAIN_DEVELOPMENT
GOBP_NEURON_MIGRATION
GOBP_SPINAL_CORD_DEVELOPMENT
GOBP_CENTRAL_NERVOUS_SYSTEM_NEURON_DIFFERENTIATION
GOBP_NEGATIVE_REGULATION_OF_NEURON_DIFFERENTIATION
GOBP_CEREBRAL_CORTEX_DEVELOPMENT
GOBP_NEUROBLAST_PROLIFERATION
GOBP_NEURAL_PRECURSOR_CELL_PROLIFERATION
GOBP_DNA_METHYLATION
GOBP_REGULATION_OF_TELOMERASE_ACTIVITY
GOBP_POSITIVE_REGULATION_OF_CELL_CYCLE_G1_S_PHASE_TRANSITION
GOBP_POSITIVE_REGULATION_OF_LIPASE_ACTIVITY
GOBP_REGULATION_OF_PHOSPHOLIPASE_ACTIVITY
GOBP_POSITIVE_REGULATION_OF_MAP_KINASE_ACTIVITY
GOBP_POSITIVE_REGULATION_OF_AUTOPHAGY
GOBP_REGULATION_OF_MAP_KINASE_ACTIVITY
GOBP_LEUKOCYTE_MIGRATION
GOBP_REGULATION_OF_LIPASE_ACTIVITY
Gene Ontology Terms
Normalized Enrichment Score
−log(FDR)
Parental origin
Androgenetic
Biparental
Parthenogenetic
F)
Paternally-expressed
Maternally-expressed
KCNQ1OT1
IGF2
FAM50B
SNRPN
INPP5F
MEST
NAP1L5
NDN
NNAT
PEG10
MAGEL2
ZDBF2
SGCE
DLK1
SLC22A3
PHLDA2
NAA60
ANO1
H19
KCNQ1
SLC22A18
CDKN1C
NLRP2
ZNF597
MEG8
MEG3
aES3_ES_1
aES3_ES_2
aES5_ES_1
aES5_ES_2
CSES7_ES_1
CSES7_ES_2
CSES2_ES_1
CSES2_ES_2
pES12_ES_1
pES12_ES_2
pES10_es_1
pES10_ES_2
PLAGL1*
CMKLR2*
RTL1*
aES3_NPC_1
aES3_NPC_2
aES5_NPC_1
aES5_NPC_2
CSES7_NPC_1
CSES7_NPC_2
CSES2_NPC_1
CSES2_NPC_2
pES12_NPC_1
pES12_NPC_2
pES10_NPC_1
pES10_NPC_2
Gene Expression Level (Z score)

Figure S1. **Additional characterization of uniparental and biparental hESCs during neural differentiation.**

A-C) PCA plots demonstrating biological replicates of the transcriptome of uniparental and biparental cells of both undifferentiated hESCs and NPCs (A), only undifferentiated hESCs (B) and only NPCs (C). D) Gene ontology (GO) enrichment analysis of genes differentially expressed between undifferentiated hESCs and NPCs for uniparental and biparental hESCs. Shown are the top 10 enriched and depleted GO terms in biparental cells. E) Flow cytometry analysis of NPCs stained with NCAM1 antibody and a control sample stained with only a secondary antibody. F) Heatmaps representing expression in uniparental and biparental hESCs of known imprinted genes that exceed TPM of 1 in either hESCs (left) or NPCs (right. Asterisk marks genes that are expressed only in NPCs). Shown are biological replicates.

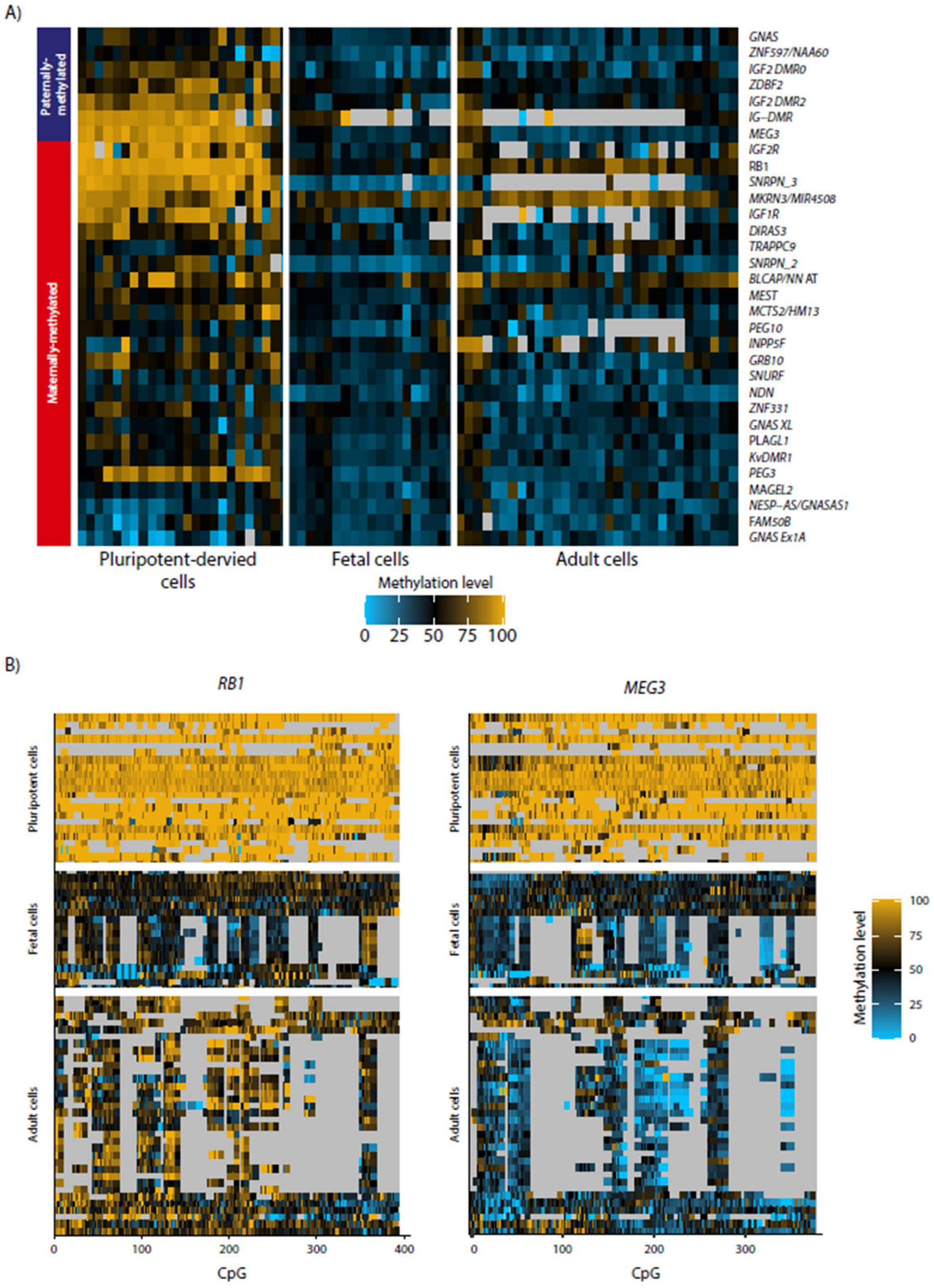
A)
Paternally-methylated
Maternally-methylated
GNAS
ZNF597/NAA60
IGF2 DMR0
ZDBF2
IGF2 DMR2
IG-DMR
MEG3
IGF2R
RB1
SNRPN_3
MKRN3/MIR4508
IGF1R
DIRAS3
TRAPPC9
SNRPN_2
BLCAP/NN AT
MEST
MCTS2/HM13
PEG10
INPP5F
GRB10
SNURF
NDN
ZNF331
GNAS XL
PLAGL1
KvDMR1
PEG3
MAGEL2
NESP-AS/GNASAS1
FAM50B
GNAS Ex1A
Pluripotent-dervied cells
Fetal cells
Adult cells
Methylation level
0 25 50 75 100
B)
RB1
MEG3
Pluripotent cells
Fetal cells
Adult cells
0 100 200 300 400
CpG
0 100 200 300
CpG
Methylation level
100 75 50 25 0

Figure S2. **Consistent DNA methylation aberrations in known imprinted loci in biparental cells.**

A) Heatmap showing the average DNA methylation of the known imprinted loci across 81 samples divided into cells that originate from pluripotent cells, fetal cells and adult cells (gray indicates NA). B) Heatmaps showing the DNA methylation levels across 81 samples of unique CpGs in RB1 imprinted locus (left) and MEG3 imprinted locus (right; gray indicates NA).

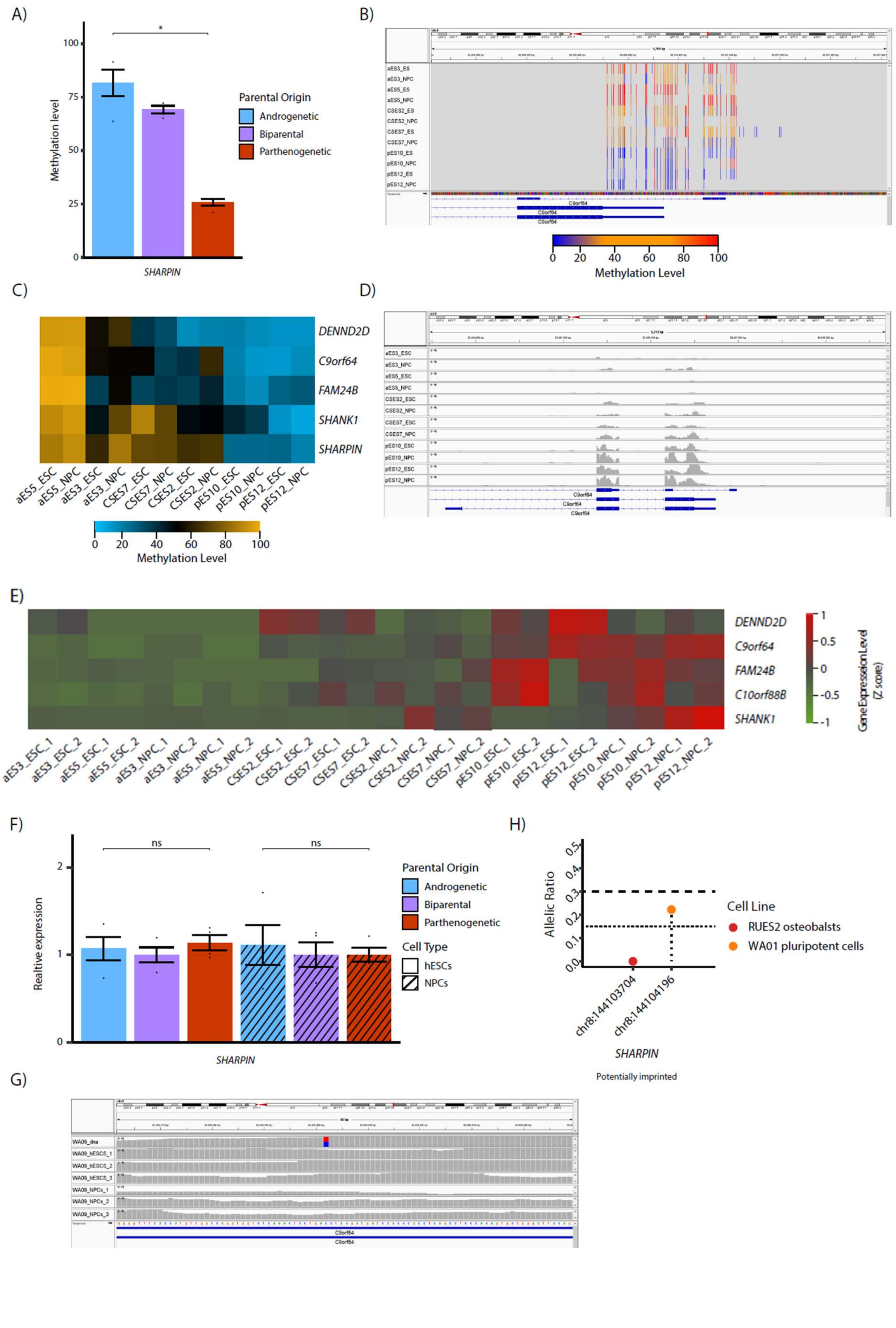
A)
*
100
75
50
25
0
Methylation level
SHARPIN
Parental Origin
Androgenetic
Biparental
Parthenogenetic
B)
aES3_ES
aES3_NPC
aES5_ES
aES5_NPC
CSES2_ES
CSES2_NPC
CSES7_ES
CSES7_NPC
pES10_ES
pES10_NPC
pES12_ES
pES12_NPC
C9orf64
0 20 40 60 80 100
Methylation Level
C)
DENND2D
C9orf64
FAM24B
SHANK1
SHARPIN
aES5_ESC
aES5_NPC
aES3_ESC
aES3_NPC
CSES7_ESC
CSES7_NPC
CSES2_ESC
CSES2_NPC
pES10_ESC
pES10_NPC
pES12_ESC
pES12_NPC
0 20 40 60 80 100
Methylation Level
D)
aES3_ESC
aES3_NPC
aES5_ESC
aES5_NPC
CSES2_ESC
CSES2_NPC
CSES7_ESC
CSES7_NPC
pES10_ESC
pES10_NPC
pES12_ESC
pES12_NPC
C9orf64
E)
DENND2D
C9orf64
FAM24B
C10orf88B
SHANK1
1
0.5
0
-0.5
-1
Gene Expression Level (Z score)
aES3_ESC_1
aES3_ESC_2
aES5_ESC_1
aES5_ESC_2
aES3_NPC_1
aES3_NPC_2
aES5_NPC_1
aES5_NPC_2
CSES2_ESC_1
CSES2_ESC_2
CSES7_ESC_1
CSES7_ESC_2
CSES2_NPC_1
CSES2_NPC_2
CSES7_NPC_1
CSES7_NPC_2
pES10_ESC_1
pES10_ESC_2
pES12_ESC_1
pES12_ESC_2
pES10_NPC_1
pES10_NPC_2
pES12_NPC_1
pES12_NPC_2
F)
ns
ns
2
1
0
Realtive expression
SHARPIN
Parental Origin
Androgenetic
Biparental
Parthenogenetic
Cell Type
hESCs
NPCs
H)
0.5
0.4
0.3
0.2
0.1
0.0
Allelic Ratio
chr8:144103704
chr8:144104196
SHARPIN
Potentially imprinted
Cell Line
RUES2 osteobalsts
WA01 pluripotent cells
G)
WA09_dna
WA09_hESCS_1
WA09_hESCS_2
WA09_hESCS_3
WA09_NPCs_1
WA09_NPCs_2
WA09_NPCs_3
C9orf64

Figure S3. **Identification of imprinted genes using uniparental and biparental hESCs and NPCs.**

A) Barplot showing the average DNA methylation levels of the *SHARPIN* promoter (mean ± SEM values, n = 4, *FDR < 0.05, Wilcoxon test). B) Browser image of the methylation values overlapping the *C9orf64* promoter. C) Heatmap showing the average DNA methylation of the promoter of the putative imprinted genes. D) Browser image depicting transcript levels of the *C9orf64* gene. E) Heatmap showing the expression of putative imprinted genes in uniparental and biparental samples that are significantly differentially expressed between the uniparental samples. F) Barplot displaying relative expression levels of *SHARPIN*, normalized to the corresponding biparental cell types, in hESCs and NPCs (mean ± SEM values, n = 4, ns = not significant). G) Genome browser view of DNA and gene expression tracks for WA09 cells at the *C9orf64* locus. Show are three samples for each cell type. H) Allelic ratio of SNP expression of *SHARPIN* gene. Points below the dotted line represent monoallelic expression. Points between the dotted and the dashed lines represent partially monoallelic expression.

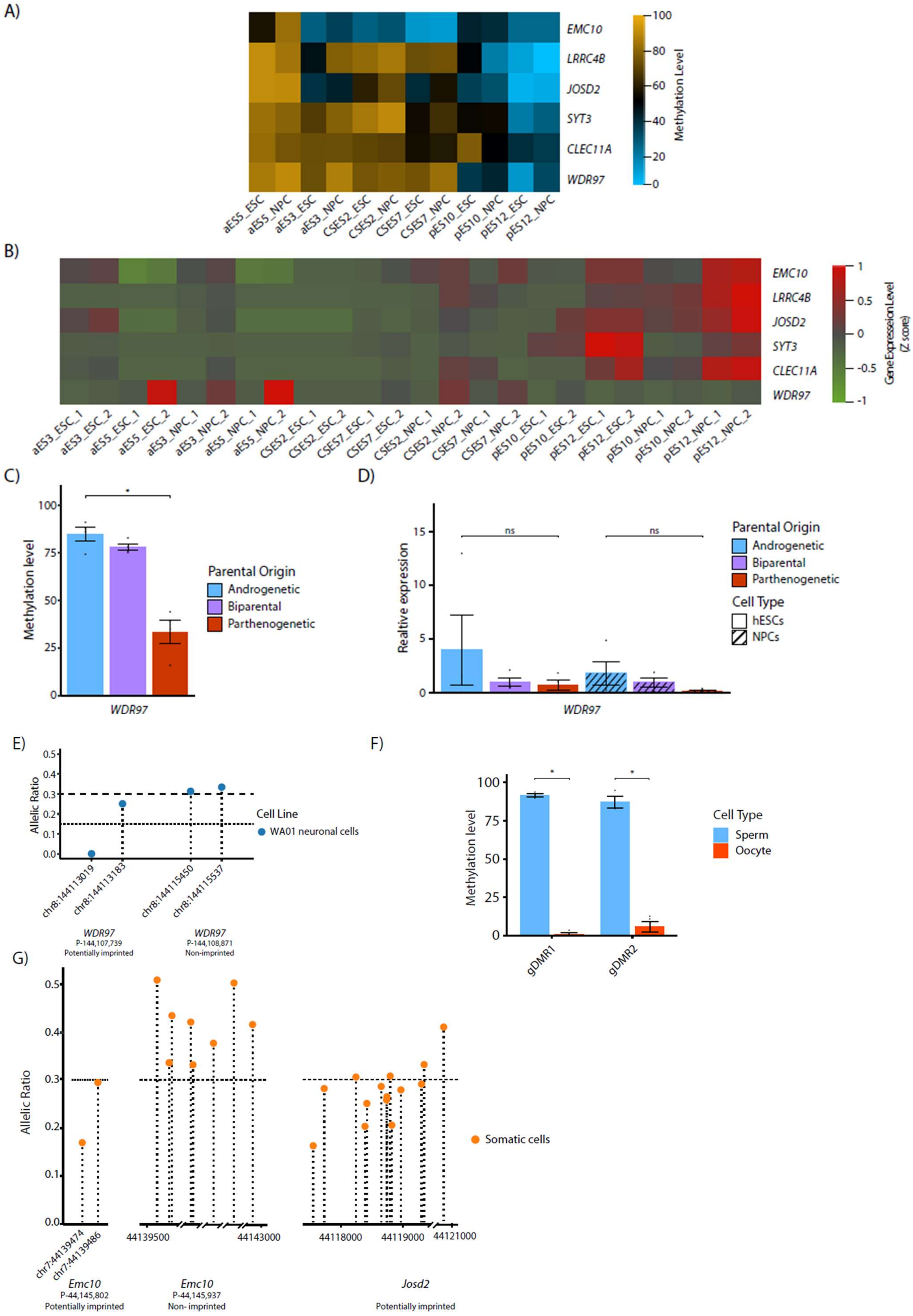

A)
EMC10
LRRC4B
JOSD2
SYT3
CLEC11A
WDR97
Methylation Level
100
80
60
40
20
0
aES5_ESC
aES5_NPC
aES3_ESC
aES3_NPC
CSES2_ESC
CSES2_NPC
CSES7_ESC
CSES7_NPC
pES10_ESC
pES10_NPC
pES12_ESC
pES12_NPC
B)
EMC10
LRRC4B
JOSD2
SYT3
CLEC11A
WDR97
Gene Expression Level (Z score)
1
0.5
0
-0.5
-1
aES3_ESC_1
aES3_ESC_2
aES5_ESC_1
aES5_ESC_2
aES3_NPC_1
aES3_NPC_2
aES5_NPC_1
aES5_NPC_2
CSES2_ESC_1
CSES2_ESC_2
CSES7_ESC_1
CSES7_ESC_2
CSES2_NPC_1
CSES2_NPC_2
CSES7_NPC_1
CSES7_NPC_2
pES10_ESC_1
pES10_ESC_2
pES12_ESC_1
pES12_ESC_2
pES10_NPC_1
pES10_NPC_2
pES12_NPC_1
pES12_NPC_2
C)
Methylation level
100
75
50
25
0
*
WDR97
Parental Origin
Androgenetic
Biparental
Parthenogenetic
D)
Realtive expression
15
10
5
0
ns
ns
WDR97
Parental Origin
Androgenetic
Biparental
Parthenogenetic
Cell Type
hESCs
NPCs
E)
Allelic Ratio
0.5
0.4
0.3
0.2
0.1
0.0
chr8:144113019
chr8:144113183
chr8:144115450
chr8:144115537
Cell Line
WA01 neuronal cells
WDR97
P-144,107,739
Potentially imprinted
WDR97
P-144,108,871
Non-imprinted
F)
Methylation level
100
75
50
25
0
*
*
gDMR1
gDMR2
Cell Type
Sperm
Oocyte
G)
Allelic Ratio
0.5
0.4
0.3
0.2
0.1
0.0
chr7:44139474
chr7:44139486
44139500
44143000
44118000
44119000
44121000
Somatic cells
Emc10
P-44,145,802
Potentially imprinted
Emc10
P-44,145,937
Non- imprinted
Josd2
Potentially imprinted

Figure S4. **Identification of imprinted locus at chromosome 8.**

A) Heatmap showing the average DNA methylation of the promoter of the putative imprinted genes on chromosome 19. B) Heatmap showing the expression of putative imprinted genes on chromosome 19 in uniparental and biparental samples. C) Barplot showing the average DNA methylation levels of the *WRD97* promoter (mean ± SEM values, n = 4, *FDR < 0.05, Wilcoxon test). D) Barplot displaying relative expression levels of *WRD97*, normalized to the corresponding biparental cell types, in hESCs and NPCs (mean ± SEM values, n = 4, ns = not significant). E) Allelic ratio of SNP expression of *WRD97*. Points below the dotted line represent monoallelic expression. Points between the dotted and the dashed lines represent partially monoallelic expression. Note that *WRD97* gene has an isoform that is potentially imprinted and an isoform that is not imprinted. F) Barplot showing the average DNA methylation levels of the genome are in mice that is syntenic to the suggested germline DMRs in chromosome 19 in human (n = 3 for sperm, n=4 for oocyte, *FDR < 0.05, obtained by permutation test). G) Allelic ratio of SNP expression in mice of two of the putative imprinted genes. Points below the dashed line represent partially monoallelic expression. Note that for *Emc10* there is an isoform that is potentially imprinted and an isoform that is not imprinted (n=3).

Table S2: List of the putative imprinted genes and their gene ID.

Putative imprinted gene	Gene ID	Putative imprinted isoform ID
DENND2D	ENSG00000162777	
SHARPIN	ENSG00000179526	
WDR97	ENSG00000179698	ENST00000323662
C9orf64	ENSG00000165118	
C10orf88B	ENSG00000255624	
FAM24B	ENSG00000213185	
EMC10	ENSG00000161671	ENST00000334976
JOSD2	ENSG00000161677	
LRRC4B	ENSG00000131409	
SYT3	ENSG00000213023	ENST00000593901
SHANK1	ENSG00000161681	
CLEC11A	ENSG00000105472	
